# Evaluation of the General Organization of Veterinary Services control program of animal brucellosis in Egypt: An outbreak investigation of brucellosis in buffalo

**DOI:** 10.14202/vetworld.2018.748-757

**Published:** 2018-06-06

**Authors:** H. I. Hosein, Hoda Mohamed Zaki, Nesreen Mohamed Safwat, Ahmed M. S. Menshawy, Sherin Rouby, Ayman Mahrous, Bahaa El-deen Madkour

**Affiliations:** 1 Department of Veterinary Medicine, Faculty of Veterinary Medicine, Beni-Suef University, Beni-Suef 62511, Egypt; 2 Department of Brucella Researches, Animal Health Research Institute, Giza, Egypt; 3 Department of Pathology, Faculty of Veterinary Medicine, Beni-Suef University, Beni-Suef 62511, Egypt; 4 General Organization of Veterinary Services, Egypt; 5 Department of Animal Medicine, Faculty of Veterinary Medicine, Aswan University, Egypt

**Keywords:** bruce-ladder, brucellosis, buffalo, histopathology, polymerase chain reaction

## Abstract

**Background and Aim:**

Brucellosis is a major constraint to livestock production in Egypt as well as many developing countries worldwide. Bovine brucellosis is an economically important disease with reproductive failure as a principal manifestation resulting in abortion, premature birth and decreased milk production in females, and orchitis and epididymitis in males. In spite of the efforts of Egyptian veterinary services to overcome brucellosis, the disease is still prevalent in both animals and humans and represents one of the most important public health hazards in Egypt. The aim of the present work was to investigate the efficacy of the control program implemented by the General Organization of Veterinary Services in *Brucella* infected buffalo farm on serological, molecular, cultural, and histopathological basis. *Brucella melitensis* biovar 3 was recovered from 6 buffalo-cows.

**Materials and Methods:**

Blood samples were collected from a total of 750 non-vaccinated lactating buffalo-cows. These animals were proved positive for *Brucella* by the Egyptian brucellosis national program. Sera were tested using buffered acidified plate antigen test and rose Bengal test as screening tests and complement fixation test as a confirmatory test. Positive animals were separated for slaughtering under the supervision of the Egyptian veterinary authorities. Remaining animals were tested every 3 weeks with slaughtering of positive cases and this continued until the remaining animals revealed three successive negative serological tests. Different lymph nodes (prescapular, prefemoral, mediastinal, retropharyngeal, and supramammary) were collected from 11 *Brucella* seropositive buffalo-cows slaughtered after being confirmed serologically as *Brucella* infected cases. Samples were collected and processed for bacterial isolation and nucleic acid detection using polymerase chain reaction (PCR). Parts of these specimens were fixed in 10% neutral buffered formalin for 48 h then processed by paraffin embedding technique.

**Results:**

“Test and slaughter” policy was applied on *Brucella* infected dairy buffalo farm. The program continued for 6 months with slaughtering of positive cases until the herd was proved *Brucella* free. *B. melitensis* biovar 3 could be recovered from six buffalo-cows. Universal PCR confirmed *Brucella* on genus level and Bruce-ladder multiplex, PCR confirmed the presence of *B. melitensis* on the species level. Histopathological examination of *Brucella*-infected lymph nodes revealed massive rarified and depleted lymphoid areas of both sub-capsular and deep cortical lymphoid follicles, macrophage cells granulomatous reaction, as well as fat, infiltrates, and chronic vasculitis. The chronic nature of *Brucella* lesions has been confirmed in this study as indicated by the chronic vasculitis and collagen deposition.

**Conclusion:**

Freedom status from brucellosis in this study required 6 months which are considered long time allowing the spread of infection to other localities especially under unhygienic conditions, husbandry system favoring mixed populations of different ages, sex, aborted and pregnant, and lack of controlled movement of animals. Therefore, effective control of animal brucellosis requires surveillance to identify infected animal herds, elimination of the reservoirs, and vaccination of young heifers. *B. melitensis* biovar 3 is the cause of the *Brucella* outbreak in buffalo which still remains the prevalent type of *Brucella* in Egypt. The disease runs a chronic course allowing further spread of infection.

## Introduction

Brucellosis is a major constraint to livestock production in Egypt as well as many developing countries worldwide. Bovine brucellosis is an economically important disease with reproductive failure as a principal manifestation resulting in abortion, premature birth and decreased milk production in females, and orchitis and epididymitis in males [1]. The disease is caused by a Gram-negative facultative intracellular bacterium capable of surviving within macrophages and leukocytes. This protects it from the humoral and cellular activities [2-5].

*Brucella* infection may occur through the gastrointestinal tract, conjunctiva, respiratory mucosa, or skin from where it spreads to local lymph nodes in which the bacteria multiply intracellularly in phagocytes followed by the invasion of lymphatic vessels and bacteremia leading to systemic infection [6]. Pathologically acute lymphadenitis is associated with burying of brucellae in lymph nodes as a point of entry [7]. The changes in the regional nodes take some weeks to fully develop, and continue for a prolonged period causing chronic lesion with the formation of histiocytic granuloma, especially in medullary sinuses. These granulomas are diffused with infiltration of giant cells [8].

Brucellosis in ruminants is most symptomatic only in primary infections where animals do not continually abort, however, aborted material, uterine, and vaginal discharges, as well as milk, contain a huge number of bacteria [9].

Bovine abortion is less common with an infection caused by *Brucella* *melitensis* than in *Brucella abortus* infections [10]. Interestingly, in Egypt *B. melitensis* is the most predominant type isolated from animals and humans [11-15].

Different *Brucella* species are incriminated as highly pathogenic in humans, with *B. melitensis* classically reported as the agent responsible for most of the human infections [16].

Direct laboratory methods of diagnosis of brucellosis in animals such as bacterial isolation have high specificity but are time-consuming and require an appropriate degree of biosafety. Polymerase chain reaction (PCR) has been introduced as an effective tool for rapid detection and confirmation of *Brucella* infection as well as differentiating *Brucella* species. Only, both culture and molecular techniques can definitively establish the presence of infection and determine the responsible *Brucella* species. On the other hand, serological procedures are used more often as quick and less expensive diagnostic tools. However, serological tests have several limitations concerning specificity and sensitivity, especially when testing individual animals [17].

Control of brucellosis presents considerable difficulties due to its contagiousness, uncertainties about detection [18], wide host range, significant numbers of carriers [19], and latently infected animals [9]. National high committee of zoonoses of the General Organization of Veterinary Services (GOVS) established the brucellosis control program which depends mainly on the test-and-slaughter policy. Herds containing even one positive animal were kept under quarantine, and all animals were to be subjected to periodical testing every 21 days. Quarantine measures were released if the animals pass three consecutive negative tests at 21 days intervals [20].

In spite of the efforts of Egyptian veterinary services to overcome brucellosis since 1988, the disease is still prevalent in both animals and humans and represents one of the most important public health hazards in Egypt as the prevalence of animal brucellosis in Egypt in the past 20 years ranged from O.33% to 1.32 % according to the official reports of the GOVS, Egypt [21].

The aim of the present work was to investigate the efficacy of the control program implemented by the GOVS in *Brucella* infected buffalo farm on serological, molecular, cultural, and histopathological basis.

## Materials and Methods

### Ethical approval

All clinical samples in this study were collected as per standard sample collection procedure without giving any stress or harm to the animals. The present work was approved by the Ethical Committee for Medical Research at the College of Veterinary Medicine, Beni-Suef University and Animal Care Guidelines of the General Organization for Veterinary Services, Egypt.

### Study area

The study was conducted on a buffalo farm of 952 buffaloes at Cairo - Ismailia desert road, Ismailia Governorate, Egypt from September 2016 to April 2017. This farm was located under quarantine by the veterinary authorities due to *Brucella* infection.

### Study population and samples

- This study was carried out on a *Brucella* infected dairy buffalo farm of 952 animals under quarantine of the GOVS. Animals were kept overcrowded and reared in open system in which animals in different ages; aborted and pregnant ones; males and females were housed together.
- Blood samples were collected from a total of 750 non-vaccinated adult lactating buffalo-cows out of 952 animals. About 20 ml of blood were aseptically drawn from jugular vein into a sterile evacuated test tube using a jack. The test tubes were left at room temperature in a sloping position to allow clotting for 2 h. The collected samples were labeled, identified and transferred to the laboratory where they held in refrigerator till the next day to give a chance for serum to separate. The serum was siphoned off by Pasteur Pipette after centrifugation at 3000 rpm for 10 min. Clear sera were stored in Cryotubes at −20°C until its use for serological studies.
- Sera were screened using buffered acidified plate antigen test (BPAT) and rose Bengal test (RBT). Positive samples were further tested with complement fixation test (CFT) as recommended by the OIE [22]. Positive animals were separated for slaughtering under the supervision of the Egyptian veterinary authorities. Animals were tested every 3 weeks for detection of animals which are still incubating the disease according to the roles of the GOVS with slaughtering of positive animals after each test. This continued until obtaining three successive negative serological tests.
- Different lymph nodes (prescapular, prefemoral, mediastinal, retropharyngeal, and supramammary) were collected from 11 slaughtered seropositive buffalo-cows. Samples were collected and processed for bacterial isolation according to Alton *et al*. [23]. Parts of these specimens were fixed in 10% neutral buffered formalin for 48 h then processed by paraffin embedding technique.

### Brucella strains

- Reference *Brucella* strains; *B. melitensis* Ether, *B. abortus* 544, and *Brucella suis* 1330 were supplied by the Central Veterinary Laboratory, Weybridge, Surrey KT15 3NB, UK.
- Field isolate of *B. melitensis* biovar 3 isolated from cattle (Collection culture of the Department of Veterinary Medicine, Beni-Suef University) was also used.
- *B. abortus* S19 vaccine strain was obtained from Veterinary Serum and Vaccine Research Institute, Abbasia, Cairo, Egypt. Reference *Brucella* strains and the field *Brucella* isolate were used as control positives for the identification and typing of *Brucella* isolates recovered in this study.

### Primers

Primer sequences used for universal and multiplex Bruce-ladder PCR are listed in Table-1.

**Table-1:** Primer sets used for PCR.

Primer	Sequence (5’–3’)	Amplicon size (bp)	DNA targets
BMEI0535f	GCG-CAT-TCT-TCG-GTT-ATG-AA	450	Immunodominant antigen, gene *bp26*
BMEI0535r	CGC-AGG-CGA-AAA-CAG-CTA-TAA		
BMEII0428f	GCC-GCT-ATT-ATG-TGG-ACT-GG	587	Erythritol catabolism, gene *eryC* (D-erythrulose-1-phosphate dehydrogenase)
BMEII0428r	AAT-GAC-TTC-ACG-GTC-GTT-CG		
BMEI0998f	ATC-CTA-TTG-CCC-CGA-TAA-GG	1682	Glycosyltransferase, gene *wboA*
BMEI0997r	GCT-TCG-CAT-TTT-CAC-TGT-AGC		
BMEII0843f	TTT-ACA-CAG-GCA-ATC-CAG-CA	1071	Outer membrane protein, gene *omp31*
BMEII0844r	GCG-TCC-AGT-TGT-TGT-TGA-TG		
BR0953f	GGA-ACA-CTA-CGC-CAC-CTT-GT	272	ABC transporter binding protein
BR0953r	GAT-GGA-GCA-AAC-GCT-GAA-G		
BMEI0752f	CAG-GCA-AAC-CCT-CAG-AAG-C	218	Ribosomal protein S12, gene *rpsL*
BMEI0752r	GAT-GTG-GTA-ACG-CAC-ACC-AA		

PCR=Polymerase chain reaction

### Serological examination

BPAT using buffered acidified plate test antigen (killed *B. abortus* strain 99 antigen, at concentration of 11% in lactate buffer, pH 3.7±0.03), RBT using RBT antigen (Rose Bengal stained, 8% cells killed *B. abortus* strain 99 antigens in lactate buffer, pH 3.65±0.05) and CFT, warm micro technique using CFT antigen (*B. abortus* biovar 1 strain 1119-3 cells in phenol saline, at a concentration of 4.5%, pH 6.8) were conducted according to Alton *et al*. [23] and OIE [22].

### Bacteriological examination

Lymph nodes of 11 seropositive buffalo-cows that were slaughtered under supervision of the GOVS were cultured on tryptose agar medium (Oxoid) with antibiotics selective antibiotic supplement [24] according to Alton *et al*. [23]. Plates were incubated at 37°C in an atmosphere of 10% CO_2_ and examined daily for 10 days for growth. The criteria used for identification were the colonial morphology, biochemical tests, requirement for additional atmospheric 10% CO_2_, production of hydrogen sulfide gas, production of urease, growth on media containing the inhibitory dyes thionin and fuchsin, agglutination with polyclonal monospecific antisera A and M and R, and phage typing using Tbilisi (Tb) and Izatnagar (Iz_1_) according to the methods recommended by Alton *et al*. [23] and OIE [22].

### DNA extraction

#### From Brucella culture

Few colonies were harvested and suspended in 200 μl of sterile, DNase, RNase-free deionized water in Eppendorf tubes. Bacterial cells were inactivated by heating the tubes at 100°C for 10 min. Killed bacterial cells were centrifuged at 15,700 g for 10 min. The supernatant containing crude DNA template was pipetted into new sterile Eppendorf tubes discarding these Diment, Ouahrani-Bettache *et al*. [25].

#### From tissue specimens

Twenty-five mg of fresh animal tissue was transferred into a microcentrifuge tube and 200 μl of GST Buffer and 20 μl of Proteinase K were added and then vortex thoroughly then incubated at 60°C overnight or until the sample lysate becomes clear. The tubes were centrifuged for 2 min at 12,000 rpm. then the supernatant was carefully transferred to a new 1.5 ml microcentrifuge tube. Extraction was carried out according to the instruction manual of gSyn^c^™ DNA extraction kit, Geneaid (New Taipei City, 22180 Taiwan, Cat. No. GS 100).

### PCR assay

Universal PCR was performed for molecular identification of *Brucella* spp. in DNA extracts from *Brucella* cultures and lymph nodes at the genus level according to Garcia-Yoldi *et al*. [26] using Primer sequences targeting Erythritol catabolism, gene *eryC* (D-erythrulose-1-phosphate dehydrogenase), and Primer sequences for amplification of target gene (Immunodominant antigen, gene *bp26*) (Table-1). On the other hand, Bruce-ladder multiplex PCR was carried out for molecular identification of *Brucella* in DNA extracts from *Brucella* cultures at the species level using five primers, table according to Garcia-Yoldi *et al*. [26], using IN gene Bruce-ladder, IN gene Bruce-ladder VR: Batch No 180515, Ingenasa, Madrid, Spain (Table-1). The PCR amplification was carried out using Labnet^®^ Multigene Gradient thermal cycler, Catalog TC9600-G- 230V (Labnet International, Inc. Edison, NJ, USA). The cycling conditions were 4 min at 95°C for initial heating, 35 cycles of 45 s at 94°C, 45 s at 60°C, 60 s at 72°C, and final extension for 7 min at 72°C. The PCR amplicons were analyzed by running 10 μl of the PCR products in 1% agarose gel stained with ethidium bromide (0.5μg/mL). Thereafter, gels were photographed under UV illumination using gel documentation and analysis system. *Brucella* species was determined according to the molecular size of the amplified products using DNA ladder (100 bp and 1 kb), (Biomatik R Code No. M7123 and M7508), Biomatik Corporation, Ontario, Canada.

*B. melitensis* positive control was used as control positive in universal PCR. *B. abortus* RB51Bruce-ladder kit control, *B. suis* Bruce-ladder kit control, *B. melitensis* Rev1 Bruce-ladder kit control, and *B. melitensis* positive control were used as positive control in Bruce ladder PCR.

### Pathological examination

Lymph nodes of all the bacteriologically positive six buffalo-cows from which *Brucella* isolated were fixed in 10% neutral buffered formalin for about 2 days. The specimens were processed by paraffin embedding method, sectioned 5-7 µm and stained with hematoxylin and eosin according to Bancroft and Gamble [27] for histopathological examination. In addition, Masson’s trichrome stain was used to confirm the presence of collagen fibers.

### Statistical analysis

Statistical analysis was carried out using the 95% confidence interval for the population proportion according to Petrrie and Watson [28].

## Results

Serological examination using BPAT, RBT, and CFT of 750 buffalo-cows revealed a prevalence of 79 (10.53%) in the first examination followed by 11 (1.64%), 52 (7.88%), 8 (1.32%), and 3 (0.50%) at the 2^nd^, 3^rd^, 4^th^, and 5^th^ examinations, respectively (Table-2). Successive serological testing continued every 3 weeks for detection of animals which are still incubating the disease with slaughtering of positive animals detected after each testing according to the roles of the GOVS. The first negative test for the remaining animals (N 597) was at the 6^th^ examination followed by the 7^th^ and 8^th^ negative examinations where the herd was considered free from *Brucella* infection. Quarantine measures were released because animals passed three consecutive negative tests at 21 days intervals. A total of 153 (20.4%) serologically positive animals were detected in this study. This program continued for about 6 months until all positive cases were detected and eliminated by slaughtering.

**Table-2:** Successive serological examinations of *Brucella*-infected buffalo farm.

Examination	Number of examined buffaloes	Positive (%)	95% CI
1^st^ (September 24)	750	79 (10.53)	(0.083, 0.126)
2^nd^ (October 16)	671	11 (1.64)	(0.006, 0.025)
3^rd^ (November 8)	660	52 (7.88)	(0.058, 0.098)
4^th^ (November 30)	608	8 (1.32)	(0.004, 0.022)
5^th^ (December 23)	600	3 (0.50)	(-0.004, 0.010)
6^th^ (January 16)	597	0 (0.00)	0
7^th^ (February 8)	597	0 (0.00)	0
8^th^ (March 4)	597	0 (0.00)	0

Total serologically positive animals 153 (20.4%). CI=Confidence interval

Bacteriological examination of different lymph nodes of 11 seropositive buffalo-cows revealed isolation of 6 (54.55%) *Brucella* isolates. Cultures were smooth, and colonies were elevated, transparent, convex, with intact borders, and brilliant surface and have a honey color under transmitted light. According to the results of biochemical tests, requirement for additional atmospheric 10% CO_2_, production of hydrogen sulfide gas, production of urease, growth on media containing the inhibitory dyes thionin and fuchsin, agglutination with polyclonal monospecific antisera A and M and R, and phage typing using Tb and Izatnagar (Iz_1_) summarized in Table-3, all the six isolates were typed as *B. melitensis* biovar 3 (Table- 3).

**Table-3:** Typing of six *Brucella* isolates recovered from buffaloes at species and biovar level.

*Brucella* isolates	CO_2_	H_2_S	Urease	Growth on dyes				Lysis by phage			Monospecific sera			Conclusion
		
				Thionin		Fuchsin		Tp		Iz_1_	A	M	R	
			
				A	b	*A*	*b*	RTD	RTD 10^4^	RTD				
Field isolates (6)	-	-	+ in 20 h	+	+	+	+	-	-	+	+	+	-	*B. melitensis 3*
Reference strains														
*B. melitensis Ether*	-	-	+ in 18-24 h	+	+	+	+	-	-	+	+	+	-	*B. melitensis 3*
*B. abortus544*	-	+	+ in 2 h	-	-	+	+	+	+	+	+	-	-	*B. abortus 1*
*B. suis1330*	-	+++	++ in <15 min	+	+	-	-	-	+	+	+	-	-	*B. suis 1*

RTD=Routine test dilution, Tp=Tbilisi, Iz_1_=Izatnagar, a=1:50000, b=1:100000, A=Anti *Brucella abortus,* M:=Anti *Brucella melitensis*, R=Rough *Brucella antiserum*

### PCR assays

**Figure-1:**
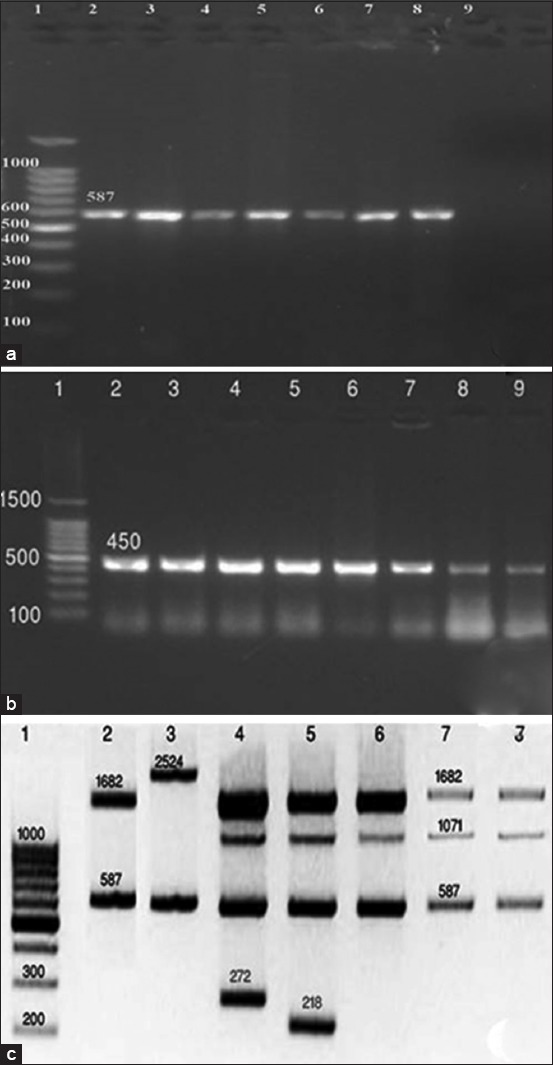
(a) Universal polymerase chain reaction (PCR) using primer sequences targeting Erythritol catabolism, gene *eryC* (Lane 1:100 bp DNA ladder, Lane 2, 3, 4, 5: *Brucella* cultures (587 bp), Lane 6: *Brucella* lymph node DNA extract (587 bp), Lanes 7, 8: *Brucella melitensis* positive control (587 bp), Lane 9: *Brucella* abortus S19 control). (b): Universal PCR using primer sequences targeting immunodominant antigen, gene *bp26* (Lane 1: 100 bp DNA ladder, Lane 2, 3, 4, 5, 6: *Brucella* cultures (450 bp), Lane 7: *B. melitensis3* culture positive control (450 bp), Lane 8, 9: *Brucella* lymph node DNA extract, 450 bp). (c): Multiplex Bruce-ladder PCR of *Brucella* isolates (Lane 1: DNA ladder, Lane 2: *B. abotus* Bruce-ladder kit control (587 bp and 1682 bp), Lane 3: *B. abortus* RB51 Bruce-ladder kit control (587 bp and 2524 bp), Lane 4: *Brucella suis* Bruce-ladder kit control (272 bp, 587 bp, 1071 bp, and 1682 bp), Lane 5: *B. melitensis Rev1* Bruce-ladder kit control (218 bp, 587 bp, 1071 bp, and 1682 bp), Lane 6: *B. melitensis* positive control (587 bp, 1071 bp, and 1682 bp), Lanes 7, 8: *B. melitensis* cultures (587 bp, 1071 bp, and 1682 bp).

Using primer sequences targeting Erythritol catabolism, gene *eryC* (D-erythrulose-1-phosphate dehydrogenase), PCR has amplified the fragment 587 bp, Figure-1a confirming the presence of *Brucella* on genus level. Using primer sequences targeting immunodominant antigen, gene *bp26*PCR has amplified the fragment 450 bp, Figure-1b confirming the presence of *Brucella* on genus level. Bruce-ladder multiplex PCR has amplified three fragments of 587 bp, 1071 bp, and 1682 bp, sizes confirming the presence of *B. melitensis* (Figure-1c). On the other hand, Bruce-ladder PCR of *B. melitensis* Rev1vaccine strain (reference Kit) amplified four fragments of 218 bp, 587 bp, 1071 bp, and 1682 bp sizes (Figure-1c). Rev1 strain only revealed the 218 bp, produced by the BMEI0752 primer pair. This primer pair detects a unique point mutation in the *rpsL* gene, coding for the ribosomal protein S12 of the vaccine strain *B. melitensis* Rev-1 and responsible for streptomycin resistance of Rev-1 [29]. *B. suis* (reference Kit) amplified fragments of 272 bp, 587 bp, 1071 bp, and 1682 bp sizes (Figure-1c) and *B. abortus* Bruce-ladder kit control amplified fragments of 587 bp and 1682 bp sizes (Figure-1c). *B. abortus* RB51 vaccine strain (reference Kit) showed two amplicons of 587 bp and the specific additional band 2524 bp (Figure-1c) which confirms the results reported by López–Goni *et al*. [30] who stated that *B. abortus* RB51 can be distinguished by a specific additional 2,524-bp fragment.

**Figure-2:**
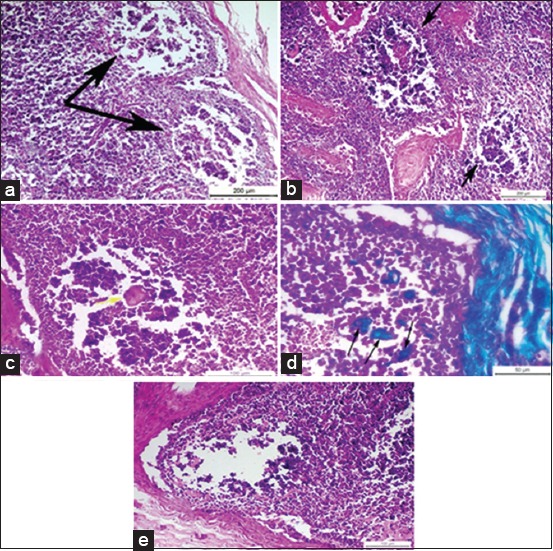
(a) Photomicrograph of a buffalo lymph node showing necrotic depleted and rarified sub-capsular lymphoid follicles, two black arrows (H and E, Bar=200 µm), (b) photomicrograph of buffalo lymph node showing multiple, necrotic, depleted, and rarified deep cortical lymphoid follicles near to corticomedullary junction, two black arrows (H and E, Bar=200 µm), (c) photomicrograph of a buffalo lymph node showing single eosinophilic structure less mass replacing the necrotic area of lymphoid follicle, yellow arrow (H and E, Bar=100 µm), (d) photomicrograph of a buffalo lymph node showing multiple green colored structure less masses of collagen, three black arrows (Masson’s trichrome, Bar=50 µm), (e) photomicrograph of a buffalo lymph node showing total lack of the germinal center of the lymphoid follicle (H and E, Bar=100 µm).

**Figure-3:**
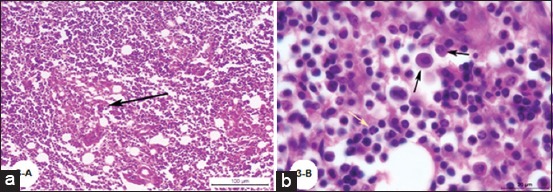
(a) Photomicrograph of a buffalo lymph node showing focal macrophage cell granulomatous reaction surrounds a focal area of necrosis (H and E; Bar=100 µm), (b) Photomicrograph of a buffalo lymph node showing diffuse macrophage cell granulomatous reaction, black arrows and presence of few numbers of neutrophils, yellow arrow (H and E; Bar=20 µm).

**Figure-4:**
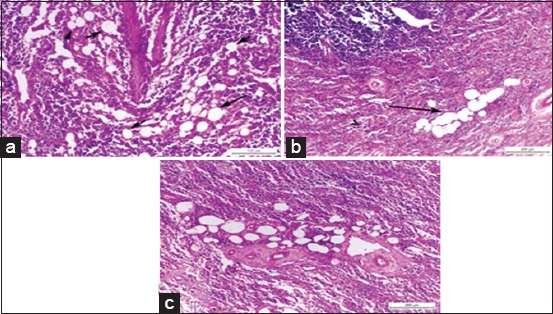
(a) Photomicrograph of a buffalo lymph node showing multiple discrete variables sized fat globules in corticomedullary junction, black arrows (H and E, Bar=100 µm), (b) photomicrograph of a buffalo lymph node showing coalescence of fat globules into a single large globule, black arrow (H and E, Bar=200 µm), (c) photomicrograph of a buffalo lymph node showing multiple fat globules around blood vessels in the deep medulla (H and E, Bar=200 µm).

**Figure-5:**
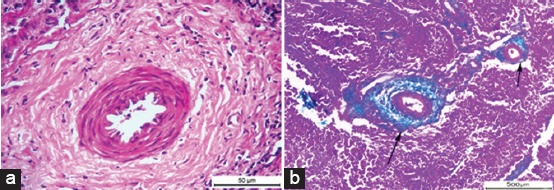
(a) Photomicrograph of a buffalo lymph node showing chronic vasculitis characterized by endothelial hyperplasia and hypertrophy in tunica media and connective tissue proliferation (H and E, Bar = 50 µm). (b) Photomicrograph of a buffalo lymph node showing green colored connective tissue around blood vessels, two black arrows (Masson’s trichrome; Bar = 500 µm).

**Figure-6:**
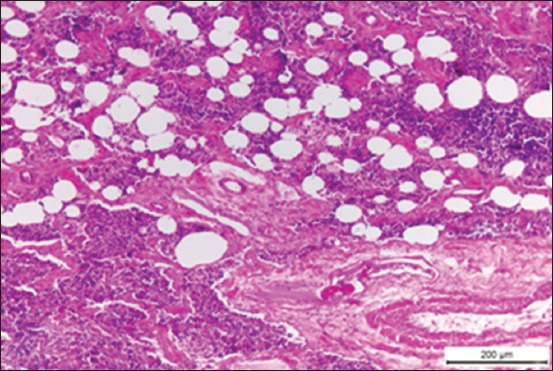
Photomicrograph of a buffalo lymph node showing perivascular edema as well as different sized fat globules (H and E, Bar=200 µm).

Histopathological examination of *Brucella*-infected lymph nodes revealed massive rarified and depleted lymphoid areas of both sub-capsular and deep cortical lymphoid follicles (Figure-2a-e). Few numbers of neutrophils, large number of lymphocytes associated with histiocytes or macrophages in the form of diffuse loose granulomatous reaction with lack of giant cells were observed (Figure-3a and b). Fatty infiltrates detected in Brucella infected lymph nodes, either discrete multiple variable sized fat globules or single large globule (Figure-4a-c). Endothelial hyperplasia and hypertrophy of tunica media and adventitia of lymphoid medullary blood capillaries with diffuse collagen deposition either in a diffuse manner or localized around blood capillaries as well as few numbers of neutrophils were observed (Figure-5a). Massive areas of necrosis were replaced by single and/or multiple eosinophilic structures less masses using hematoxylin and eosin and green in color using Masson’s trichrome stain till the total lack of the germinal center (Figure-5b). Perivascular edema (Figure-6) could be detected.

## Discussion

The seroprevalence of anti-*Brucella* antibodies in 750 buffalo-cows using BPAT, RBT, and CFT was 10.53% (n=79) in the first examination. Such high prevalence may be due to exposure of the highly susceptible non-vaccinated buffalo-cows to *Brucella* infection through the introduction of an infected animal which is considered an extremely important risk factor that can lead to spread of the infection to the whole herd. Removal of infected animals for slaughtering failed to stop the spreading of the disease, as the serotesting continued to detect positive animals in the subsequent testing for over 6 months where at last 597 buffalo-cows proved serologically negative for three successive tests with 3 weeks intervals. This can be attributed to a husbandry system favoring mixed populations of different ages, sex, aborted and pregnant, and lack of calf hood vaccination. Animals incubating the disease and latent infections as it has been estimated by Nielsen and Duncan [3] that about 20% of calves born by infected heifers could be found persistently infected with brucellae may play a role in the failure of the control program. In addition, exposure of susceptible animals to uterine and vaginal discharges of infected animals that contain a huge number of bacteria is an important risk factor for disease transmission between infected and susceptible animals as reported by Holt *et al*. [31]. Infected tissues and milk are the main sources of environmental contamination that spreads the disease horizontally [9]. Of extremely important consideration is the possible long interval between *Brucella* infection and development of clinical signs. It is reasonable to say that the current results indicate an outbreak of *Brucella* infection. Efforts of the veterinary authorities to control brucellosis in Egypt through the organized “test and slaughter program” failed to eliminate the disease or reduce the prevalence of the disease as the prevalence of animal brucellosis in Egypt in the past 20 years ranged from 0.33%-1.32 % according to the official reports of the GOVS, Egypt [21]. Nearly similar prevalence was recorded by Abd-El Halim *et al*. [19] in Assiut, Egypt who recorded the prevalence of the disease in buffaloes as 12.9% with failure of control program of the GOVS. Lack of surveillance program to identify infected animals/herds and absence of control of the movement of infected animals to prevent re-introduction of the disease which is considered the major risk factor, especially in Egypt [26], are contributing factors.

Eradication of brucellosis by test and slaughter seems unfeasible in developing countries because of limited resources to compensate farmers whose animals are slaughtered during such screening programs [32].

*Brucella* organisms were isolated from different lymph nodes of six seropositive buffalo-cows out of 11. Failure of isolation from the other 5 positive serological cases may be due to a low number of viable *Brucella* organisms in the tissue sample or due to contamination with other bacteria and the fastidious nature of *Brucella* organisms [33]. Typing of the six *Brucella* isolates involved a combination of growth characteristics including colonial morphology, oxidase, urease, CO2 requirement, H2S production, growth in the presence of fuchsin and thionin, lysis by Tbilisi and Izatnagar, Iz_1_ bacteriophages and agglutination with monospecific A and M anti-sera that confirmed the presence of *B. melitensis* biovar 3.

*B. melitensis* biovar 3 was considered as the prevalent *Brucella* type in Egypt [11-15]. In addition, Abd-El Halim *et al*. [19] in Assiut, Egypt, isolated the same brucella species and biovar isolated in this study; *B. melitensi*s biovar 3 from 3 aborted buffaloes out of 87 (3.45%). Primarily, *B. melitensis* infects small ruminants. Transmission to large ruminants may be the outcome of close contact between sheep, goats, cattle, and buffalo which is considered by Holt *et al*. [31] as the main risk factor of transmission of *B. melitensis* biovar 3 to cattle and buffalo in Egypt where *B. melitensis* biovar 3 is the prevalent type in both large and small ruminants.

Using primer sequences targeting Erythritol catabolism, gene *eryC* (Derythrulose-1-phosphate dehydrogenase), PCR has amplified the fragment 587 bp, Figure-1a confirming the presence of *Brucella* on genus level. Interestingly, the PCR testing *B. abortus* S19 DNA did not produce the 587-bp fragment common to all *Brucella* strains as it is the only *Brucella* strain unable to oxidize erythritol [34]. The preferential utilization of erythritol rather than glucose is characteristic for pathogenic *Brucella* strains [35]. Using primer sequences targeting immunodominant antigen, gene *bp26*PCR has amplified the fragment 450 bp, Figure-1b confirming the presence of *Brucella* on genus level. These assays are designed to exploit a single unique genetic locus. They are useful for identification when species is not critical [29].

Concerning molecular characterization of *Brucella* isolates on the species level, Bruce-ladder multiplex PCR has amplified three fragments of 587 bp, 1071 bp, and 1682 bp, sizes Figure-1c confirming the presence of *B. melitensis*. On the other hand, Bruce-ladder PCR of *B. melitensis* Rev1 vaccine strain has amplified four fragments of 218 bp, 587 bp, 1071 bp, and 1682 bp sizes. Rev1 strain only revealed the 218 bp, produced by the BMEI0752 primer pair. This primer pair detects a unique point mutation in the *rpsL* gene, coding for the ribosomal protein S12 of the vaccine strain *B. melitensis* Rev-1 and responsible for streptomycin resistance of Rev-1, [30]. *B. suis* has amplified fragments of 272 bp, 587 bp, 1071 bp, and 1682 bp sizes and *B. abortus* Bruce-ladder kit control has amplified fragments of 587 bp and 1682 bp sizes. *B. abortus* RB51 vaccine strain (Figure-1c) showed two amplicons of 587 bp and the specific additional band 2524 bp which confirms the results reported by López–Goni *et al*. [36] who stated that *B. abortus* RB51 can be distinguished by a specific additional 2,524-bp fragment. This can be explained after Vemulapalli *et al*. [37] who demonstrated that the *wboA* gene encoding a glycosyltransferase, an enzyme essential for the synthesis of O antigen, is disrupted by an IS711 element (an insertion sequence) in *B. abortus* RB51 and computer analysis of the nucleotide sequence from RB51 revealed that the *wboA* gene was interrupted by an 842-bp fragment that resulted in development of the 2524 fragment (1682+842=2524).

Such multiplex Bruce-ladder PCR assay proved capable to differentiate *Brucella* species as it is directed toward genetic loci that are variable among species [29]. Differential PCR-based assays are particularly useful for epidemiological traceback. The major advantage of PCR assays is the time taken compared to conventional methods which require several days to isolate and identify *Brucella* organisms [38,39].

Histopathological examination of *Brucella*-infected lymph nodes revealed massive rarified and depleted lymphoid areas of both sub-capsular and deep cortical lymphoid follicles. Similar findings were reported by Ahmed *et al*. [40] who reported lymphoid depletion in the white pulp of spleen in buffaloes naturally infected with *B. melitensis*. Moreover, María-Jesús *et al*. [41] described mild lymphoid depletion in the splenic nodules in mice experimentally infected with virulent *Brucella* organisms. On the other hand, this finding comes in contrast with Barrionuevo *et al*. [42], Jubb *et al*. [7], and Pollak *et al*. [43] who found that lymphoid hyperplasia was the most predominant feature in case of brucellosis. In addition, Xavier *et al*. [44] reported variable degrees of lymphoid hyperplasia associated with neutrophilic and histiocytic infiltrates in the medulla and paracortex in *Brucella* infected bovine internal iliac lymph nodes and Meador *et al*. [45] described lymphofollicular hyperplasia, sinusoidal histiocytosis, and medullary plasmacytosis in bovine supramammary lymph nodes. Interestingly, Neta *et al*. [46] reported that *Brucella* infection resulted in lymphoid hyperplasia in bovine lymph nodes and spleen, and lymphoid depletion in the thymus and Hosein *et al*. [47] reported hyperplasia in the white pulp of the spleen of most examined *Brucella* infected cows and depletion in the white pulp of spleen and the cortex of lymph nodes of some cows. Cheville *et al*. [48] explained lymphoid depletion as a result of immunodeficiency due to the multiplication of *Brucella* in the lymphocytes and macrophages of infected animals. The obtained results revealed very few numbers of neutrophils, a large number of lymphocytes associated with histiocytes or macrophages in the form of a diffuse loose granulomatous reaction with lack of giant cells. Such results have been supported by those of Barrionuevo *et al*. [42] and Pollak *et al*. [43] who attributed the presence of a population of monocytes to the secondary internalization of the bacteria.

The chronic nature of brucellosis has been confirmed in this study as indicated by the chronic vasculitis that was characterized by endothelial hyperplasia and hypertrophy of tunica media and adventitia of lymphoid medullary blood capillaries with diffuse collagen deposition either in a diffuse manner or localized around blood capillaries, as well as few numbers of neutrophils.

Chronic granulomatous lymphadenitis observed in this study, coincide with the results obtained by Benitez *et al*. [49] who mentioned that collagen fibrotic proliferation could contribute to granuloma formation as the compact structure of granuloma successfully prevents the dissemination of the microorganisms. Perivascular edema may suggest the second episode of bacterial invasion to the tissue especially when associated with chronic lesions. Fatty infiltrates detected in Brucella infected lymph nodes, either discrete multiple variables sized fat globules or single large globule, may be attributed to gradual replacement of the lymph nodes parenchyma with fat. Similar findings were reported by Gonzales-Peramato *et al*. [50].

## Conclusion

Freedom status from brucellosis in this study required 6 months which are considered long time allowing the spread of infection to other localities especially under unhygienic conditions, husbandry system favoring mixed populations of different ages, sex, aborted and pregnant, and the lack of controlled movement of animals. Therefore, effective control of animal brucellosis requires surveillance to identify infected animal herds, elimination of the reservoirs and vaccination of young heifers. *B. melitensis* biovar 3 is the cause of the *Brucella* outbreak in buffalo which still remains the prevalent type of *Brucella* in Egypt. The disease runs a chronic course allowing further spread of infection.

## Authors’ Contributions

HIH designed the study, interpreted the results and reviewed the manuscript. AM performed the fieldwork and collected the samples. HMZ was responsible for the bacteriological identification of the *Brucella* isolates. AMSM, SR and BEM carried out the PCR assays and drafted the manuscript NMS was responsible for the histopathological examination. All authors read and approved the manuscript.

## References

[1] Corbel M.J (2006). Brucellosis:An overview. Emerg. Infect. Dis.

[2] Ackermann M.R, Cheville N.F, Deyeoe B.L (1988). Bovine ileal dome lymphoepithelial cell:Endocytosis and transport of *Brucella abortus* strain 19. Vet. Pathol.

[3] Nielsen K.H, Duncan J.R (1990). Animal Brucellosis.

[4] Riley L.K, Robertson D.C (1984). Ingestion and intracellular survival of *Brucella abortus* in human and bovine polymorphonuclear leukocytes. Infect. Immun.

[5] Silva F.L, Paixa o, T.A, Borges A.M, Lage A.P, Santos R.L (2005). Bovine brucellosis. Cad. Tecn. Vet. Zootecnia.

[6] Ko J, Splitter G.A (2003). Molecular host-pathogen interaction in brucellosis:Current understanding and future approaches to vaccine development for mice and humans. Clin. Microbiol. Rev.

[7] Jubb K.V.F, Kennedy P.C, Palmer N (2016). Pathology of Domestic Animals.

[8] Khoudair R.M, Ibrahim E.M, Saker G.G, Hafez M.A (2009). Clinico-diagnostic and pathological studies on cattle and buffaloes suffering from brucellosis and tuberculosis in Kafr El Sheikh Governorate. Egypt. J. Comp. Path. Clin. Pathol.

[9] Radostits O.M, Gay C.C, Hinchcliff K.W, Constable P.D (2007). Diseases associated with *Brucella* species. Veterinary Medicine:A Textbook of the Diseases of Cattle, Horses, Sheep, Pigs and Goats.

[10] Olsen S, Tatum F (2010). Bovine brucellosis. The veterinary clinics of North America. Food Anim. Pract.

[11] Hosein H.I, Rouby S, Menshawy A, Ghazy N (2016). Seroprevalence of camel brucellosis and molecular characterization of *Brucella melitensis* recovered from dromedary camels in Egypt. Res. J. Vet. Pract.

[12] Hosein H.I, Rouby S.R, Menshawy A, AbdAl-Ghany A.E (2017). Sensitivity and specificity of the commonly used diagnostic procedures of bovine brucellosis. Vet. Sci. Res. Rev.

[13] Affi M.M, Abdul-Raouf U.M, El-Bayoumy E.M, Montasser A.M, Mohamad H.A (2015). Isolation and biotyping of *Brucella melitensis* from Upper Egypt. Report Opin.

[14] Menshawy A, Perez-Sancho M, Garcia-Seco T, Hosein H.I, Garcia N, Martinez I, Sayour A.E, Goyache J, Azzam R.A.A, Dominguez L (2014). Assessment of genetic diversity of zoonotic *Brucella* spp. Recovered from livestock in Egypt using multiple locus VNTR analysis. BioMed. Res. Int.

[15] Salem A.A, Hosein H.I (1990). *Brucella* strains prevalent in Egypt. Assuit. Vet. Med. J.

[16] Pappas G, Akritidis N, Bosilkovski M, Tsianos E (2005). Brucellosis. N. Eng. J. Med.

[17] Kaltungo B, Saidu S, Sackey A, Kazeem H (2014). A review on diagnostic techniques for brucellosis. Afr. J. Biotechnol.

[18] Fensterbank R (1986). Brucellosis in cattle, sheep and goats:Diagnosis, control and vaccination. Rev. Sci. Tech. Off. Int. Epiz.

[19] Abd-El Halim M.H, Abeer A.E, Shalaby M.N.A (2017). Prevalence of brucellosis in buffaloes and its control measures. J. Vet. Med. Res.

[20] Refai M (2002). Incidence and control of brucellosis in the Near East region. Vet Microbiol.

[21] Wareth G, Hikal A, Refai M, Melzer F, Roesler U, Neubauer H (2014). Animal brucellosis in Egypt. J. Infect. Dev. Ctries.

[22] OIE (2016). Brucellosis *Brucella abortus, B. melitensis* and *B. suis* Chapter 2.1.4.

[23] Alton G.G, Jones L.M, Angus R.D, Verger J.M (1988). Techniques for Brucellosis Laboratory Institute.

[24] Ewalt D.R, Packer R.A, Harris S.K (1983). An improved selective medium for isolating *Brucella* sp. from bovine milk. Proceeding of the Process International Symposium Veterinary Laboratory Diagnosticians.

[25] Ouahrani-Bettache S, Soubrier M.P, Liautard J.P (1996). IS6501-anchored PCR for the detection and identification of *Brucella* species and strains. J. Appl. Bacteriol.

[26] Garcia-Yoldi D, Marin C.M, De Miguel M.J, Munoz P.M, Vizmanos J.L, Lopez-Goni I (2006). Multiplex PCR assay for the identification and differentiation of all *Brucella* species and the vaccine strains *Brucella abortus* S19 and RB51 and *Brucella melitensis* Rev1. Clin. Chem.

[27] Bancroft J.D, Gamble M (2008). Theory and Practice of Histological Techniques.

[28] Petrrie A, Watson P (2013). Statistics for Veterinary and Animal Science.

[29] Bricker B.J (2002). PCR as a diagnostic tool for brucellosis. Vet. Microbiol.

[30] Cloeckaert A, Vizcaino N, Paquet J.Y, Bowden R.A, Elzer P.H (2002). Major outer membrane proteins of *Brucella* spp.:Past, present and future. Vet. Microbiol.

[31] Holt H, Eltholth M, Hegazy Y, El-Tras W, Tayel A, Guitian J (2011). *Brucella* spp. infection in large ruminants in an endemic area of Egypt:Cross-sectional study investigating seroprevalence, risk factors and livestock owner's knowledge, attitudes and practices (KAPs). BMC Public Health.

[32] Godfroid J, Scholz H, Barbier T, Nicolas C, Wattiau P, Fretin D, Whatmore A, Cloeckaert A, Blasco J, Moriyon I, Saegerman C, Muma J.B, AL Dahouk S, Neubauer H, Letesson J.J (2011). Brucellosis at the animal/ecosystem/human interface at the beginning of the 21^st^century. Prev. Vet. Med.

[33] Seleem M.N, Boyle S.M, Sriranganathan N (2010). Brucellosis:A re-emerging zoonosis. Vet. Microbiol.

[34] Rodríguez M.C, Viadas C, Seoane A, Sangari F.J, López-Goñi I, García-Lobo J.M (2012). Evaluation of the effects of erythritol on gene expression in *Brucella abortus*. PLoS One.

[35] Sangari F.J, Aguero J, Garcia-Lobo J.M (2000). The genes for erythritol catabolism are organized as an inducible operon in *Brucella abortus*. Microbiology.

[36] Lopez-Goni I, Garcia-Yoldi D, Marin C.M, de Miguel M.J, Munoz P.M, Blasco J.M, Jacques I, Grayon M, Cloeckaert A, Ferreira A.C, Cardoso R, Correa de Sa M.I, Walravens K, Albert D, Garin-Bastuji B (2008). Evaluation of multiplex PCR assay (Bruce-ladder) for molecular typing of all *Brucella* species including the vaccine strains. J. Clin. Microbiol.

[37] Vemulapalli R, McQuiston J.R, Schurig G.G, Sriranganathan N, Halling S.M, Boyle S.M (1999). Identification of an IS711 element interrupting the wboA gene of *Brucella abortus* vaccine strain RB51 and a PCR assay to distinguish strain RB51 from other *Brucella* species and strains. Clin. Diagn. Lab. Immunol.

[38] Bricker B.J, Halling S.M (1994). Differentiation of *Brucella abortus*1, 2, and 4*Brucella melitensis*
*Brucella ovis* and *Brucella suis* biovar 1 by PCR. J. Clin. Microbiol.

[39] Ewalt D.R, Bricker B.J (2000). Validation of the abbreviated *Brucella* AMOS PCR as a rapid screening method for differentiation of *Brucella abortus* field strain isolates and the vaccine strains 19 and RB51. J. Clin. Microbiol.

[40] Ahmed Y.F, Sokkar S.M, Desouky H.M, Madbouly A.A (2012). Studies on buffalo-cows naturally infected with *Brucella melitensis*. Glob. Vet.

[41] María-Jesús G, Blasco J.M, Gorvel J.P, Moriyón I, Moreno E (2012). What have we learned from brucellosis in the mouse model?. Vet Res.

[42] Barrionuevo P, Delpino M.V, Velasquez LN, Samartino C.G, Coria L.M, Ibanez A.E, Rodriguez M.E, Cassataro J, Giambartolomei G.H (2011). *Brucella abortus* inhibits IFN-gamma-induced FcgammaRI expression and FcgammaRI-restricted phagocytosis via toll-like receptor 2 on human monocytes/macrophages. Microbes Infect.

[43] Pollak C.N, Delpino M.V, Fossati C.A, Baldi P.C (2012). Outer membrane vesicles from *Brucella abortus* promote bacterial internalization by human monocytes and modulate their innate immune response. PLoS One.

[44] Xavier M.N, Paixa˜o T.A, Poester F.P, Lage A.P, Santos R.L (2009). Pathological, immunohistochemical and bacteriological study of tissues and milk of cows and fetuses experimentally infected with *Brucella abortus*. J. Comp. Pathol.

[45] Meador V.P, Deyoea L, Chevill N.D.N.F (1989). Pathogenesis of *Brucella abortus* infection of the mammary gland and supramammary lymph node of the goat. Vet. Pathol.

[46] Neta C, Alcina V, MoAl J.P.S, Xavier M.N, Paixão T.A, Lage A.P, Santos R.L (2010). Pathogenesis of bovine brucellosis. Vet J.

[47] Hosein H.I, EL-Nahass E.L.S, Rouby S.R, El-Nesr K.A (2018). Detection of *Brucella* in tissues and in formalin-fixed paraffin-embedded (FFPE) specimens using PCR. Adv. Anim. Vet. Sci.

[48] Cheville N.F, Olsen S.C, Jensen M.G, Stevens A.M (1996). Bacterial persistence and immunity in goats vaccinated with a pur E deletion mutant or the parenteral 16M strain of *Brucella melitensis*. Infect. Immun.

[49] Benitez p.C.A, Serantes D.R, Herrmann C.K, Viglietti A.I.P, Vanzulli S, Giambartolomeia G.H, Comercib D.J, Delpino M.V (2016). The effector protein bpe005 from *Brucella abortus* induces collagen deposition and matrix metalloproteinase 9 downmodulation via transforming growth factor β1 in hepatic stellate cells. Infect Immunol.

[50] Gonzalez-Peramato P, Jimenez-Heffernan J.A, Sabater C, Vicandi B (2002). Lipogranulomatous lymphadenopathy as a potential source of error in fine needle aspiration cytology. A case report. Acta Cytol.

